# ModuleDigger: an itemset mining framework for the detection of *cis*-regulatory modules

**DOI:** 10.1186/1471-2105-10-S1-S30

**Published:** 2009-01-30

**Authors:** Hong Sun, Tijl De Bie, Valerie Storms, Qiang Fu, Thomas Dhollander, Karen Lemmens, Annemieke Verstuyf, Bart De Moor, Kathleen Marchal

**Affiliations:** 1 Department of Electrical Engineering, Katholieke Universiteit Leuven, Kasteelpark Arenberg 10, 3001 Leuven, Belgium; 2 Department of Engineering Mathematics, university of Bristol, Bristol BS8 1TR, UK; 3 Department of Microbial and Molecular systems, Katholieke Universiteit Leuven, Kasteelpark Arenberg 20, 3001 Leuven, Belgium; 4 Laboratory for experimental medicine and endocrinology, Katholieke Universiteit Leuven, 3000 Leuven, Belgium

## Abstract

**Background:**

The detection of *cis*-regulatory modules (CRMs) that mediate transcriptional responses in eukaryotes remains a key challenge in the postgenomic era. A CRM is characterized by a set of co-occurring transcription factor binding sites (TFBS). *In silico *methods have been developed to search for CRMs by determining the combination of TFBS that are statistically overrepresented in a certain geneset. Most of these methods solve this combinatorial problem by relying on computational intensive optimization methods. As a result their usage is limited to finding CRMs in small datasets (containing a few genes only) and using binding sites for a restricted number of transcription factors (TFs) out of which the optimal module will be selected.

**Results:**

We present an itemset mining based strategy for computationally detecting *cis*-regulatory modules (CRMs) in a set of genes. We tested our method by applying it on a large benchmark data set, derived from a ChIP-Chip analysis and compared its performance with other well known *cis*-regulatory module detection tools.

**Conclusion:**

We show that by exploiting the computational efficiency of an itemset mining approach and combining it with a well-designed statistical scoring scheme, we were able to prioritize the biologically valid CRMs in a large set of coregulated genes using binding sites for a large number of potential TFs as input.

## Background

In eukaryotic genomes transcriptional regulation is often mediated by the concerted interaction of several transcription factors and cofactors [1]. Each transcription factor recognizes its own binding site or regulatory motif. The combination of several transcription factor specific motifs is called a *cis*-regulatory module (CRM). The presence of a *cis*-regulatory module thus determines the transcriptional response of a specific gene. As coexpression might imply a similar mechanism of coregulation, coexpressed genes can be searched for the presence of statistically overrepresented CRMs. Some strategies have been developed to search *de novo *for the best transcription factor binding site combination, such as for instance CisModule [2]. The complex nature of the problem, however, still poses some restrictions on the applicability of these *de novo *algorithms. Most of the more pragmatic module detection methods are combinatorial search strategies that start from a set of binding sites for individual motifs. These binding sites are obtained by screening intergenic sequences with each TF-specific position weight matrix (PWM). Subsequently these methods search for the motif combination that is statistically most overrepresented in a set of genes of interest, as compared to the background [3-6]. Although these algorithms can in principle be applied to sets of coexpressed genes, most of them do not explicitly assess the specificity of the overrepresented module for the observed expression pattern in the coexpressed geneset. Exceptions are for instance CREME [4], which provides an extensive statistical framework and ModuleMiner [7], which does apply a leave one out strategy in combination with a genomewide ranking to define the modules most specific for the coexpressed geneset as compared to the remainder of the genome. The drawback of the latter method is that the underlying optimization procedure is computationally very intensive restricting its use to relatively small sets of genes and a small number of TFs.

In this work we propose a novel framework for the detection of CRMs based on itemset mining. We tested our framework on a benchmark compiled from a recent ChIP-Chip experiment in human stem cells and compared its performance to several previously described module detection algorithms.

## Results

### Module detection framework

**Figure 1:**
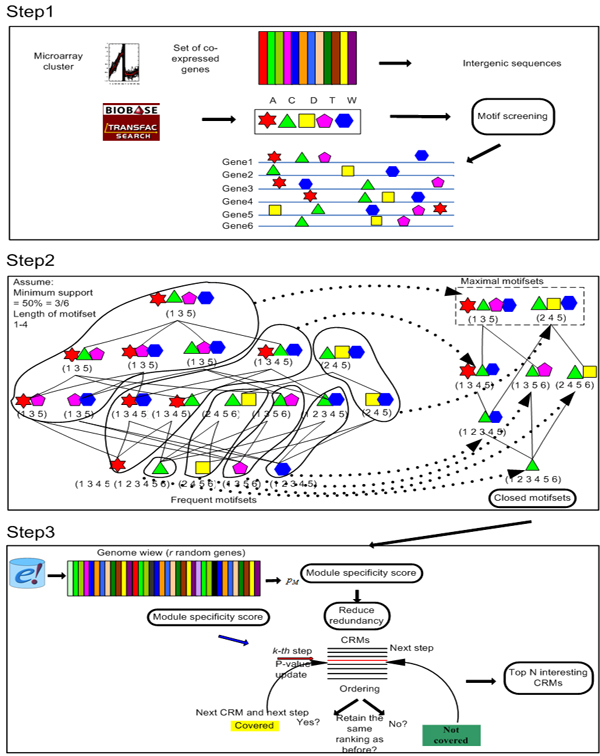
**Analysis flow**. The input consists of a set of coexpressed or coregulated genes. Step 1: Screening the intergenic sequences of these genes with a library of PWMs. Step 2: Apply our itemset mining strategy to find all the modules (closed motifsets) that occur in a minimal number of genes in the dataset (a minimum support defined by the user). Step 3: Determine the final rank of each module. An original ranking is assigned to each module based on the *module specificity score*. In the update step the rank of overlapping modules is reduced by iteratively assigning an updated score.

The analysis flow we used is outlined in Figure 1. Like other module detection methods, our method starts from an existing library of PWMs extracted from TRANSFAC [8] (step 1). All intergenic sequences of a coexpressed or coregulated set of genes are screened with those PWMs to identify per PWM the p-value of the best hit in each sequence [9]. The search for CRMs then boils down to searching through an exponentially large number of combinations of these individual binding sites (step 2). Traditional optimization based methods rely on heuristics to make this search computationally tractable; however, such methods come with no guarantee that a globally optimal solution will be found. In contrast, here we applied a strategy from itemset mining [10] (see Methods). Itemset mining approaches exhaustively investigate all possibly interesting solutions (in this case, motif modules or CRMs), and hence do not suffer from local optima problems. They are able to do this despite the exponential number of combinations of binding sites by exploiting properties of the search space that allow for efficient pruning during the search. The output of our itemset mining algorithm is an exhaustive list of all possible motif modules (or potential CRMs). To filter the biologically most interesting CRM candidates from this list, we compute a score for each of the potential CRMs (see Methods). This score assess how specific this CRM is for the set of genes in which it occurs, and for the cluster of input genes as a whole. A CRM is considered significant for the genes in which it occurs if that geneset does not contain many other overrepresented CRMs, and it is considered specific for the whole cluster of input genes if the CRM is statistically more overrepresented in this cluster of genes than in the remainder of the genome. By iteratively applying this scoring system we can prioritize a list of non-redundant and most promising CRMs. The higher the rank of a CRM in this list, the higher its potential of being a biologically valid one (as it is the most specific for the genes in which it occurs and the most explanatory for the whole set of input genes). As such, our framework combines advantages associated with the efficiency of an itemset mining search strategy with those related to statistical scoring measures.

### Benchmark dataset

The application of chromatin immunoprecipitation combined with DNA microarray techniques (ChIP-chip) in eukaryotes allows the genome wide mapping of the physical interaction between a TF and its target gene. Our test set was derived from a genomewide ChIP-Chip analysis performed by Boyer et al. 2005 [11] (see Methods). It consists of 116 genes that co-bind three core TFs, OCT4, SOX2, NANOG (involved in plurypotency and self-renewal) in their 1000 bp proximal promoter region. Moreover, the three TFs bind in each other's close proximity turning them in a true case example of a CRM. The advantage of this dataset over previous ones is that it is much larger (the muscle dataset [12], for instance contains 12 genes), which allows us to fully exploit the potential of our method.

Note that ModuleDigger will normally be applied to sets of genes that are coexpressed, as identified for example by microarray data. Here the set of genes is selected based on ChIP-chip data instead. While this may be unusual in practical applications, knowing exactly which regulators bind the intergenic regions of the set of genes selected, allows us to better assess the performance of our method.

### Modules detected by ModuleDigger on the benchmark set

**Figure 2:**
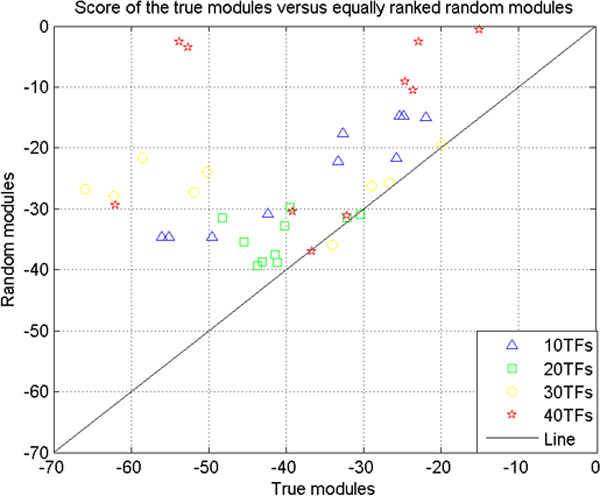
**Module scores of the valid modules versus equally ranked random modules**. For each valid module its score (log value, the lowest one is the best) is plotted versus the score (log value) of the equally ranked module in the randomized dataset. Different symbols correspond to the different datasets of increasing complexity (using respectively 10, 20, 30 and 40 TFs as input).

To test ModuleDigger we ran it on the 1000 bp proximal promoter regions of all 116 genes. As mentioned above, ModuleDigger uses a two step approach: it first exhaustively enumerates all CRMs that occur in the benchmark geneset and subsequently assigns a rank to all CRMs that is proportional to the specificity of the module for the geneset in which it occurs and for the set of input genes as a whole. For benchmarking our method we considered only the module consisting of the three TFs OCT4, SOX2 and NANOG, as a valid module. All other modules were considered biologically invalid. Note that this is a conservative assumption, which may result in an overestimation of the number of modules considered to be biologically invalid as the genes of our dataset may contain other yet uncharacterized CRMs. The performance of the algorithm is assessed by the average rank the valid module receives after running the algorithm. In our test we started off with the simple case in which we only used ten TFs as input (the three true TFs together with seven randomly sampled TFs) (see Methods for Running parameters). The complexity of the problem was increased by gradually including more randomly selected TFs (20, 30 or 40 TFs) (see Additional file 1 for the motif models from TRANSFAC and Additional file 2 for the sets of TFs). To asses the statistical significance of the ranked modules, we used a strategy described by Tusher et al. [13]. We compared the score that the valid module received with the score of a module that received a rank similar to the one of the valid module, in a randomized version of the dataset (see Methods, Figure 2). We can then conclude that if the score of the biologically valid module is higher than the score of an equally ranked module in more than 90% of the randomized datasets, it was successfully assigned a significantly high rank by ModuleDigger. We also assessed the number of false positive modules that should be expected to be discovered by ModuleDigger, by counting the number of modules in the randomized dataset that contained a score higher than the score of the true module in our benchmark dataset.

These results (Table 1) show that in the presence of a restricted number of TFs (10, 20, 30 or 40), ModuleDigger is able to identify the biologically valid module consistently and significantly as one of the more highly ranked modules. Inspecting the composition of the more highly ranked modules showed that many of them consist of (a) random TF(s) in combination with at least either OCT4, SOX2 or NANOG. Further increasing the noise in the dataset (including more random TFs not belonging to the experimentally verified CRM) lowers the rank of the biologically valid CRM and increases the number of invalid modules among the higher ranked modules. The search space increases and it becomes easier to randomly detect modules that receive a score equal to the score of the biologically valid module.

**Table 1:** Running ModuleDigger in the presence of a gradual increase in noise.

Number of TFs included												
Test set	10 TFs			20 TFs			30 TFs			40 TFs		

Runs	RANK	S	FP	RANK	S	FP	RANK	S	FP	RANK	S	FP

1	6	y	1	18	y	3	41	y	29	15	y	12

2	12	y	2	9	y	2	35	y	3	153	y	2

3	12	y	2	11	y	3	45	y	39	55	y	1

4	11	y	3	14	y	14	71	y	66	54	y	9

5	3	y	1	12	y	1	15	n	18	138	y	52

6	1	y	0	8	y	3	38	y	1	48	y	23

7	1	y	0	4	y	3	52	y	3	141	y	49

8	2	y	0	6	y	3	58	y	1	147	y	3

9	4	y	2	12	y	11	32	y	1	155	y	52

10	1	y	0	5	y	3	45	y	39	158	y	81

Average	6.3	/	1.1	10	/	4.5	43.2	/	20	108	/	28.4

Median	5	/	1	10	/	3	43	/	10.5	139	/	17.5

Std	4.7	/	1.1	4.3	/	4	15.2	/	22.6	56	/	28.0

For each specified number of TFs, 10 different runs were performed which differed in the PWMs randomly selected from TRANSFAC. Average, Median and Std: average, median and standard deviation of the rank of the biologically valid module (OCT4, SOX2 and NANOG). RANK: the rank the valid module received in our output; Score of valid module versus random modules (S): assesses whether the score of the true module is higher than the score of an equally ranked module in a randomized dataset (y = yes, n = no). Number of false positives (FP): the number of modules in the randomized dataset that contained a score higher than the score of the valid module in our benchmark dataset.

### Comparison with other CRM detection algorithms

In this section, we compare our module detection framework first with a *de novo *module detection tool CisModule [2]. Next we evaluate the performance of ModuleSearcher [3] on our benchmark data set. ModuleSearcher [3] is a module detection algorithm that starts from a library of known PWMs and subsequently searches for the combination of binding sites that is overrepresented in the set of coregulated genes. Finally, we compare ModuleDigger with Clover [6]. Although Clover was developed to search for individually over-represented TFBS in the intergenic sequences of coexpressed genes, it has previously shown to also be able to compete with module detection methods in identifying true regulatory modules.

First we ran CisModule [2], a *de novo *algorithm on our benchmark set (see methods). CisModule did detect a module of three TFs in our dataset, but none of the binding sites corresponded to the OCT4, SOX2 and NANOG binding sites. We can, however, not exclude that indeed a module composed of yet unknown sites is more overrepresented than the module described by Boyer et al. [11].

Secondly, we tested to what extent Clover [6], ModuleSearcher [3] and ModuleDigger were able to detect a module of the three known binding sites in our benchmark set (Table 2) (see Methods). We used all 116 intergenic sequences as input. The number of input PWMs was varied. We started with the easy case in which we only provided four PWMs as input, i.e. the three PWMs known to belong to the CRM and one randomly sampled from TRANSFAC (Table 2A). Then we gradually increased the complexity of the problem, i.e. finding the right CRM by using more TFs as input (Table 2B) (see Additional file 3 for the sets of TFs that were used in this analysis). For each tool we recorded the running time and tested whether the output contained the CRMs for which Boyer et al. [11] provided experimental evidence. Because not all methods could identify the module consisting of the three TFs OCT4, SOX2, and NANOG in the set of coregulated genes, we define in this section not only the module containing the three TFs as the valid module but also the modules consisting of two out of three TFS are considered to be valid modules.

**Table 2:** Comparison between methods.

**A**									
Method	Clover			ModuleSearcher(A*)			ModuleDigger		

Running time	1.6 min			0.5 min			10 s		

	NM	SRR	Sn	NM	SRR	Sn	NM	SRR	Sn

OCT4, SOX2, NANOG	0	0	0	2.4	10	1.1%	6	10	27.6%

OCT4, SOX2	3.9	10	45.3%	0	0	0	2	10	49.1%

OCT4, NANOG	0	0	0	0	0	0	3	10	42.2%

SOX2, NANOG	0	0	0	2.4	10	0.9%	9	10	28.4%

									

**B**									

Method	Clover			ModuleSearcher (A*)			ModuleDigger		

Running time	4 min			0.5 min			20 s		

	NM	SRR	Sn	NM	SRR	Sn	NM	SRR	Sn

OCT4, SOX2, NANOG	0	0	0	0	0	0	5	10	28%

OCT4, SOX2	6.8	10	45.3%	21	5	2.8%	18	10	49%

OCT4, NANOG	0	0	0	0	0	0	23	10	42%

SOX2, NANOG	0	0	0	21	4	0.5%	20	10	9%

(A) For all algorithms we used as input the benchmark set, the PWMs of OCT4, SOX2, NANOG and one random PWM. NM: number of modules present in the output for those runs where the RR = 1 (average over runs where RR = 1). SRR (summation of recovery rate): number of runs for which the output contained a module corresponding to the valid modules (OCT4, SOX2, NANOG or combinations thereof). Sn: number of genes containing the valid module in the output (average of runs for which RR was 1). (B) Similar to (A) but using OCT4, SOX2, NANOG in combination with 7 randomly selected PWMs.

When ModuleSearcher [3] was run in the A* mode and using four TFs as input, it was able to find the complete OCT4, SOX2 and NANOG module in all 10 runs with a quite high specificity. More specifically, besides the module consisting of the three TFs, the output contained on average 2.4 other modules amongst which were also the SOX2, NANOG module. The OCT4, SOX2 and NANOG module could, however, only be located in a very small number of the 116 genes (0.9%). No output was obtained when running ModuleSearcher using the genetic algorithm mode. Clover [6] detected only OCT4, SOX2 as single overrepresented TFs but not NANOG. Therefore only the combination OCT4, SOX2 was retrieved in every run and among on average 3.9 other modules. The sensitivity was higher than for ModuleSearcher: the module was present in 45.3% of the 116 genes. ModuleDigger found all valid modules (combinations of three and two motifs) in all runs, and this with a rather good specificity. For the OCT4, SOX2, NANOG module, for instance, over the 10 runs on average 6 modules were more highly ranked than the OCT4, SOX2, NANOG module. Note however, that amongst these 6 modules there are also the combinations OCT4, SOX2 (average rank 3) and OCT4, NANOG (average rank 4). The sensitivity is also quite high as valid modules were detected in 27–49% of the genes.

When repeating the experiment for ten TFs, only ModuleDigger is able to find the complete OCT4, SOX2, NANOG module and this with a very high specificity and sensitivity. From these results it is clear that it is much easier to assign a high rank to a module of three TFs than to a module of two TFs. For the valid modules that consist of two motifs, ModuleSearcher and ModuleDigger perform equally well regarding the specificity, as assessed by the total number of modules (NM) that were identified. However, ModuleDigger detects the valid modules in more test runs, as indicated by the sum of the recovery rate (SRR) over all ten runs. ModuleDigger also shows a higher sensitivity, i.e. the number of genes in which the valid modules are detected is larger.

## Discussion

Our itemset mining methodology detects CRMs by taking as input a set of genes, assuming that at least a subset of these are coregulated, and searches for a recurrent pattern of TFBS. The complete analysis flow consists of three steps: Step 1: motif screening step to predict all possible TFBS; Step 2: the enumeration of all frequent CRMs (all possible closed motifsets); Step 3 the module rank assignment (the prioritization of modules). Our method differs from previous approaches in that it first enumerates all the possible combinations of the TFBSs given in the input, and subsequently applies an iterative ranking step in which CRMs that are specific for the set of genes in which the CRM occurs (gene specificity score), statistically overrepresented in the complete set of input genes (coexpression specificity score), and not overlapping with higher ranked CRMs, are prioritized by assigning them an overall module score. By using this prioritized list of modules we can define how many modules still score higher than the one the user selects and by using order statistics we can assess the number of false positives to expect if we choose a specific threshold for the module score. The advantage of using an itemset mining approach instead of an optimization-based strategy is clear from the comparison with other module detection methods. First, the algorithm is much faster and can easily be applied to larger datasets (containing more genes and more TFs). Secondly, because it exhaustively explores all solutions it does not risk to get stuck in a local optimum. Optimization based strategies may not return any output, or they may output a module different from the biologically valid one, even if it is the global optimum.

## Conclusion

We have presented an efficient methodology to detect CRMs based on itemset mining, implemented in the tool ModuleDigger. By applying our method on a benchmark dataset of increasing complexity we have explored the potential and limits of our strategy.

## Methods

### Benchmark dataset

From the UCSC database (human assembly of NCBI 35) [14] we could retrieve a match for 333 gene names out of the 353 names originally listed as being cobound by OCT4, SOX2 and NANOG (genes names as listed in [11]). We retrieved the corresponding 1000 bp intergenic sequences of these 333 genes from UCSC. Only those sequences were retained for which the binding of OCT4, SOX2 and NANOG was located in the 1000 bp proximal promoter region. This resulted in 116 intergenic sequences known to bind OCT4, SOX2 and NANOG (see Additional file 4 for the list of 116 genes).

### Running parameters ModuleDigger

Potential binding sites for individual motifs were identified by screening the intergenic sequences of each of the genes of the benchmark data set by motif models described in TRANSFAC [8]. Screening was performed with the method of Hertzberg et al. [9]. The advantage of this method is that it converts screening scores to p-values by using a randomization strategy. Using p-values instead of raw scores allows comparing motif hits obtained with motif models that are different in length. A motif matrix is compiled by discretizing the screening results with a one indicating that the particular TF contains at least one hit of the corresponding PWM within the upstream region of the gene and a zero that it does not contain a hit. A threshold on the Hertzberg screening p-value of 0.4 was chosen.

To estimate for each TF the number of occurrences of its binding site on a genomewide level (co-expression specificity score see below), we selected 5000 random sequences with a length equal to the length of our benchmark sequences (1000 bp) (see Additional file 5 for the list of 5000 genes). Frequencies of genomewide occurrence were derived by converting the screening results to a corresponding random motif matrix, discretizing this matrix with a similar Hertzberg p-value threshold as for the test data and counting for each TF the number of occurrences (ones). The minimum support parameter of ModuleDigger, specifying the required minimum number of genes in a CRM, was set to two. For the tests outlined in Table 1, we set the parameters such that all CRMs contained exactly three motifs. For the tests outlined in table 2, we choose parameter settings such that all CRMs contain two or three motifs.

### Running parameters for other tools

We only included module detection methods for which the command line version was available in order to be able to optimize parameter settings for our dataset. EMCMODULE [5] was not included because it requires all TFBS to have the same length, a restriction that is not valid for our dataset.

ModuleSearcher was obtained from the author [15]. For ModuleSearcher we used both the genetic and A* algorithm as optimization strategy. As input we used binding site predictions obtained by screening the intergenic sequences using MotifScanner [16] with prior set as 0.2, and a 3th order background model [17]. For all parameter settings we used default settings except for the maximum number of motifs and for the length of the region within which a module should be contained. The maximum number of motifs was set to two or three motifs. For the length of the promoter region that should contain the module we used both 200 bp (default value) and 1000 bp.

Clover was downloaded from the website of the paper. The input consisted of the intergenic sequences, the PWMs, and the human background model (default background model as suggested by the author). The p-value threshold for a motif to be called significant was set to 0.05. We compiled potential modules from the output of Clover by making all combinations of at least two TFs from the TF that were found significantly enriched in our benchmark dataset. Then we checked whether the true modules were among the collection of potential modules.

For CisModule the number of motifs to search for was set to one, two or three and the length of the module was set to 200 bp and 1000 bp separately, we ran it for 1000 iterations, and set the motif length from 12 bp to 15 bp.

### Benchmarking ModuleDigger

**Figure 3:**
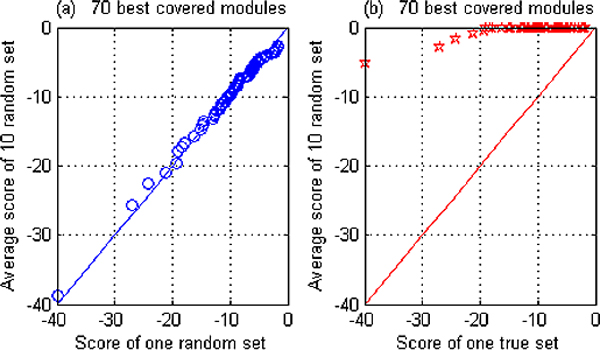
**Module scores of the valid modules versus equally ranked random modules**. Plot of the scores of the 70 best ranked modules versus a baseline for a) a random set and b) the true sets. The baseline consists of the average scores of the 70 best ranked modules in 10 different randomizations. For the "true sets" we used as input a set of 10 TFs amongst which OCT4, SOX2 and NANOG were present, while the random sets use as an input a set of TFs without the OCT4, SOX2 and NANOG. The random sets are thus not expected to contain any true modules. Panel a) all selected modules are random and reflect scores of false positives. They are distributed on the diagonal of the first quadrant. Panel b) the scores of the highly ranked modules in the true dataset score consistently higher than the equally ranked modules in the random sets.

For testing the noise sensitivity of our method we applied ModuleDigger on the benchmark dataset of 116 genes and gradually increased the number of TFs that composed the input search space. Each combination consisted of the experimentally verified TFs together with a number of noisy TFs randomly sampled from TRANSFAC. Each test was run 10 times. In each run we recorded the rank and the score of the biologically valid module, consisting of three TFs OCT4, SOX2 and NANOG. The significance of the ranking was assessed based on order statistics as described by Tusher et al. [13]. The idea behind this approach is represented in Figure 3. In a randomized set we expect the scores to be neutral, not reflecting any true signal. A randomized set is composed by searching for modules in the 116 sequences using a TF set as input which does not contain the true TFs known to be present in the data. Different modules obtained in randomized and in the real set are ranked according to their score and plotted against a baseline. The baseline is constructed by making 10 randomized sets, ranking their modules and averaging the scores of the equally ranked modules. The baseline thus consists of average scores of randomized sets. When comparing a random set with the baseline we expect all values to be close to the diagonal of the first quadrant (Figure 3a). When plotting the modules found in the non-randomized dataset against the baseline, it is clear that the highest ranked modules have a score, which is consistently better than the score of the equally ranked modules in the random set, reflecting the true signals in the real data set (Figure 3b).

### Benchmarking with other tools

For comparison with the other methods, we used for all methods the 116 intergenic sequences. Most of the previously developed module detection tools require a data reduction prior to their usage. This data reduction is usually based on preselecting regions conserved between species and the TFBS located within them. In most of the described analyses this results in sets of 500 bp in length per sequence and an input of about 20 TFs that can make up the module. For our benchmarking, we mimicked data reduction by only including for each gene the proximal promoter region and by providing a restricted number of TFs of which we knew they were present amongst the experimentally verified modules. In the comparative analysis we started with a first analysis containing four TFs (three experimentally verified ones and one sampled randomly from 584 TFs present in TRANSFAC). Each test was repeated ten times, each time randomly sampling another TF. We repeated all analyses using ten TFs as input. We assessed the results by calculating the sum of the recovery rate (SRR), the number of modules in the output (NM) and the sensitivity. The recovery rate (RR) equals one if the true module was among the results of a specific test. The SRR thus corresponds to the number of tests that contain the true modules. If the module was recovered (i.e. if RR = 1), we also computed the NM, or the number of modules that were identified (for Modulesearcher this equals the total number of modules in the output which contain at least two motifs; for Clover this equals the total number of combinations one can make with TFs called significant; for ModuleDigger this equals the number of modules ranked higher than the true module). The sensitivity is defined as the number of genes out of the 116 in which the module was detected. All values reported are averages over the 10 test runs.

### ModuleDigger algorithm

#### Enumerating all frequent closed CRMs (Figure 1 step 2)

For the identification of modules, defined as combinations of individual motifs, we rely on itemset mining. Itemset mining searches for the combination of items (in our case the motifs) that are supported by a minimal number of transactions (in our case the genes). We used an implementation provided in the package MINI which is based on CHARM [10]. CHARM searches for closed sets using a dual itemset-tidset (motifset-geneset) search tree. A closed set is a set of motifs (or a potential module) that is frequent (i.e., simultaneously contained in the intergenic region of a minimal number of genes) and that can no longer be extended by additional motifs without decreasing the number of genes with all these motifs in their intergenic region. CHARM is designed to efficiently limit the number of combinations to be tested if different itemsets (or motifsets) are related to each other by a valid "subset" relation, meaning an itemset can only satisfy all constraints if all of its subsets do. A consequence is that we can search for modules by starting with very small motifsets (containing just one motif), gradually expanding them, and stopping (or pruning) the search once a motifset is reached which does not meet a lower bound on the number of genes that contain that motifset. This pruning step results in a massive speed-up, making the method applicable to large data sets. Implementing the subset relation for the motif data is straightforward as the motif matrix is a binary matrix: a target gene has a motif instance for a regulator if the corresponding gene-regulator entry in the motif matrix is equal to one. In our set up an itemset was called valid if it contained at least two genes.CHARM outputs all possible closed motifsets (or equivalently closed CRMs). This list is exhaustive and still contains many redundant (i.e., partially overlapping modules) modules as well as modules that are not biologically interesting because they are not specifically associated with the set of genes in our benchmark set.

#### Assigning a rank to each CRM (Figure 1 step 3)

To assess the statistical significance of the selected modules we adapted the filtering strategy described in MINI [18]. The scoring scheme developed here depends on two scores outlined below: the geneset specificity score and the coexpression specificity score.

#### Geneset specificity score

For the *geneset specificity score *we assume that the initial null model considers all genes to be 'independent'. In particular, under this null hypothesis the presence of a motif in the intergenic region of a gene is assumed to be independent of its presence in the intergenic region of the other genes. For a particular gene *g*, we can estimate the probability to contain any given motif in its intergenic region as the fraction of all motifs present in its intergenic region. Formally this is $f_{g}=\frac{c_{g}}{N_{m}}$, where *c*_*g *_= *count*(*g*) is the total number of motifs that have at least one hit in gene *g *and *N*_*m *_is the total number of motifs from the used PWM library.

The independence assumption implies that the probability for a set of genes *g *∈ *G *to simultaneously contain any given motif equals *p*_*G *_= ∏_*g *∈ *G*_*f*_*g*_, i.e., the product of the probabilities for each individual gene from *G *to contain the motif. Based on *p*_*G*_, we can use the binomial distribution to compute for a specific geneset *G *the probability that it contains any *s *motifs under the null hypothesis:

(1) $$P(s,N_{m},p_{G})=\left(\begin{array}{c} N_{m}\\ s \end{array}\right)p_{G}^{s}{\left(1-p_{G}\right)}^{N_{m}-s}$$

Hence, the probability that a geneset *G *contains a motifset with at least *s *motifs is given by cumulating this probability over all values larger than or equal to *s*:

(2) $$\begin{array}{ccc} P_{c}^{G}=\sum_{t=s}^{N_{m}}\left(\begin{array}{c} N_{m}\\ t \end{array}\right)p_{G}^{t}{\left(1-p_{G}\right)}^{N_{m}-t}, & with & p_{G}=\prod_{g\in G}f_{g} \end{array}$$

The smaller $P_{c}^{G}$, the more surprising such a motifset would be under the null hypothesis. Hence, a smaller value of $P_{c}^{G}$ indicates a stronger deviation from the null hypothesis of independence, and hence that a stronger association between the motifs in the geneset *G*.

#### Coexpression specificity score

The geneset specificity score does not yet take into account the identity of the motifs contributing to a particular module. In other words, it does not assess to what extent a particular module is explanatory for the total set of input genes. Therefore we calculate a second *coexpression specificity p-value*: to this end we formulate a null hypothesis under which we assume that the motifs are the independent random variables, meaning that each motif has its own specific probability of occurrence in any given gene, independent of the presence of the other motifs in the gene. The probability of each individual motif *m *is derived from its frequency of occurrence: $f_{m}=\frac{c_{m}}{N_{g}}$ where *c*_*m *_corresponds to the number of intergenic sequences in the genome that contain at least one hit of the motif, and *N*_*g *_is the total number of background genes considered The independence assumption implies that the probability of finding a particular module (being a set of motifs *m *∈ *M*) in a gene equals *p*_*M *_= ∏_*m *∈ *M*_*f*_*m*_, the product of the individual probabilities of each of the single motifs. Using these module probabilities, the probability of finding a particular motifset *M *in a set of *s *genes out of the total cluster of *n *genes by chance can be calculated by the binomial distribution:

(3) $$P_{c}^{M}=\left(\begin{array}{c} n\\ s \end{array}\right)p_{M}^{s}{\left(1-p_{M}\right)}^{n-s}$$

The probability of finding a motifset *M *in at least *s *of the *n *genes is calculated by means of the cumulative binomial distribution function, as

(4) $$\begin{array}{cc} P_{c}^{M}=\sum_{t}^{n}\left(\begin{array}{c} n\\ t \end{array}\right)p_{M}^{t}{\left(1-p_{M}\right)}^{n-t}, & with:p_{M}=\prod_{m\in M}f_{m} \end{array}$$

Stronger deviations from the null hypothesis assuming motif independence are revealed by smaller values of $P_{c}^{M}$, which may in turn reveal an association between the genes containing the motifs in *M*.

#### Module specificity score

For each module identified by the itemset mining approach we calculate both the specificity p-value of a module for a geneset and the coexpression specificity p-value. The final score assigned to a module (*module specificity score*) is

(5) $$p-value=\max(P_{c}^{G},P_{c}^{M})$$

and (somewhat abusively) we refer to it as the p-value of the CRM. By selecting CRMs with a small p-value defined in this way, we ensure that neither of the null hypotheses discussed above can explain the association pattern revealed by the motifset and the geneset of the CRM.

#### Iterative p-value updating of the module score

We have noted that the set of closed CRMs is already a reduced representation of the set of all frequent CRMs across the set of input genes. Additionally, the *module specificity score *from Equation 5 allows us to rank CRMs in order of decreasing significance (i.e. in order of increasing p-value). However, in practice this list will still contain many partially overlapping modules. For instance, consider two CRMs which occur in almost the same genes and of which the first is composed of two motifs (M1 and M2) while the second module consists of three motifs, partially overlapping with the motifs of the first module (M1, M2, M3). It is not uncommon that both such highly redundant CRMs are highly ranked in the list of CRMs after sorting it in order of decreasing significance. To avoid the output being overwhelmed by a large number of highly redundant CRMs, we need to correct for redundancy between CRMs, and we do this by means of an iterative procedure that in each iteration selects the next most interesting CRM, conditioned on the CRMs already selected so far.

To start, the list of closed CRMs is sorted according to the *module specificity scores *calculated as in Equation 5. The CRM on top of the list is then selected as the most interesting CRM and removed from the list. To select the subsequent CRMs, an iterative procedure is applied. In each iteration, the p-values of all CRMs still in the list are adjusted, ensuring that the CRM with smallest adjusted p-value remains on top of the list. This p-value adjustment (described in detail below) is designed to penalize CRMs that overlap with already selected CRMs, in order to make sure that selected CRMs are as non-redundant as possible with the ones that have previously been selected. Subsequently, the top-ranked CRM is selected and removed from the list as well.

To explain the iteration more in detail, let us assume that *k *CRMs have already been selected, with gene sets *G*_*i *_for *i *= 1,..., *k *(i.e., we are now in iteration *k*). The set of genes of all already selected CRMs is denoted by ${\hat{G}}_{k}=\underset{i=1:k}{\cup }G_{i}$. Let *M *be a motifset of a CRM in the list of which the p-value (Equation 5) will need to be adjusted. We will discuss how the *coexpression specificity score *(Equation 4) can be adjusted; for the *geneset specificity score *(Equation 2) the procedure is analogous, and combining these two allows one to adapt the *module specificity score *from Equation 5.

All we will do is adapting the way in which *p*_*M *_is computed in Equation 4: the probability that a random gene's motifset contains all motifs from *M*. As noted earlier, all motifs from *M *can simultaneously be among a gene's motifs by pure chance, assuming independence of the motifs. However, after a few iterations, it may also be attributable to associations already identified in previous iterations by already selected CRMs. In particular, for any gene *g*, all motifs that have been part of the motifset of an already selected CRM containing *g *in its geneset, have already been associated with gene *g*. Let us denote this set of motifs for a gene *g *by *M*_*k*_(*g*). Then, we adapt *p*_*M *_in the following way (where again *N*_*g *_is the number of background genes):

(6) $$p_{M}^{'}=\frac{N_{g}-\left|{\hat{G}}_{k}\right|}{N_{g}}\left(\prod_{m\in M}f_{m}\right)+\frac{1}{N_{g}}\sum_{g\in {\hat{G}}_{k-1}}\left(\prod_{m\in M\backslash M_{k}(g)}f_{m}\right)$$

Here the first term captures the probability that all motifs from *M *are associated in a random gene, assuming that the motifs occur independently of each other. At iteration *k*, such genes are estimated to occur with a prior probability of $\frac{N_{g}-\left|{\hat{G}}_{k}\right|}{N_{g}}$, and the probability that they do contain all motifs in *M *is estimated as $\prod_{m\in M}f_{m}$. The second term captures the probability that a gene belonging to an already selected CRM contains all motifs from *M*. This probability is possibly larger than under the independence model, estimated by multiplying just those *f*_*m *_for motifs that were not part of the already selected CRMs containing the gene *g*, i.e. for motifs *m *that do not belong to *M*_*k*_(*g*).

Note that this adjusted value of *p*'_*M *_reduces to the initial definition of *p*_*M *_in Equation 4 if no CRMs have already been covered (i.e. if *k *= 0). Then the first factor in the first term is equal to one, and the second term is equal to 0. However, for larger values of *k *it can only increase in value. As a result, also the p-value as computed by Equation 4 and hence also Equation 5 can only increase. This means that we can avoid having to adjust all p-values for all CRMs still in the list, and still be able to select the CRM that is most significant after adjustment. We can do this by starting with the CRM at the top of the list, adjusting the p-value, and reinserting the CRM with adjusted p-value in the list in order to maintain the correct order. If the new position after reinsertion is still on top (i.e. its adjusted p-value is smaller than all p-values for all other CRMs, whether already adjusted or not), this means that it would remain on top even after adjusting the p-values of the lower-ranked CRMs. Hence, it can be selected as the next CRM in the output and removed from the list, thus ending the iteration.

The pseudocode of the entire iterative algorithm is given below. The set *R *of all closed CRMs is sorted according to its *module specificity score*. The most highly ranked CRM is then selected and removed from *R *(steps 1–4).

Algorithm (*R*)

1: for each *M *∈ *R *do

2:    $p-value=\max(P_{c}^{G},P_{c}^{M})$

3: sort *R *in ascending order of these p-values

4: select the top-ranked CRM from *R *and remove it from *R*

5: *k *:= 1

6: repeat until a sufficient number of CRMs are selected:

7:    *CRM *:= the top ranked CRM in *R*

8:    adjust the p-value of *CRM*

9:    insert *CRM *in *R *to keep *R *sorted in order of increasing p-value

10:    if *CRM *remains top-ranked after reinsertion:

11:       select *CRM *and remove it from *R*

12:       *k*:= *k*+1

Then the iterative updating and selection of CRMs starts. If after the updating of its p-value the CRM remains top-ranked in *R*, then that CRM is considered to be interesting and is selected (steps 7–12). The iteration counter *k *is incremented and the next iteration starts. The iteration can be stopped as soon as enough CRMs have been selected.

## List of abbreviations used

CRM: *Cis*-regulatory module; NM: Number of modules in the output; RR: Recovery Rate; SRR: Sum of the Recovery Rate; TF: Transcription factor; TFBS: Transcription factor binding site.

## Competing interests

The authors declare that they have no competing interests.

## Authors' contributions

HS, VS and KM designed the study. TDB designed and implemented the itemset mining strategy. HS, VS, KM analyzed the data. HS, TDB, VS, QF, TD, KL, MV, BDM and KM contributed materials/analysis methodology. HS, KM, TDB and KL wrote the paper.

## Supplementary Material

Additional file 1 **Motif models from TRANSFAC**. Additional file 1 is a text file that contains the motif matrices for OCT4, SOX2 and NANOG and 584 other regulators as obtained from TRANSFAC. Click here for file

Additional file 2 **The sets of 10, 20, 30, 40 regulators used for benchmarking ModuleDigger**. Additional file 2 is a text file. Additional file 2 contains the regulator names that were selected in addition to the three regulators OCT4, SOX2 and NANOG but also the regulator names that were used for construction of the completely random sets. These data sets were used as input for ModuleDigger. Click here for file

Additional file 3 **The sets of 4 and 10 regulators used for comparing ModuleDigger with other CRM detection tools**. Additional file 3 is a text file. Additional file 3 contains the regulator names that were selected in addition to the three regulators OCT4, SOX2 and NANOG. These data sets were used as input for ModuleDigger and other CRM module detection tools. Click here for file

Additional file 4 **List of 116 genes**. Additional file 4 is a text file that contains the gene names of the 116 genes that were retrieved from the 353 genes originally listed as being cobound by OCT4, SOX2 and NANOG [11]. Click here for file

Additional file 5 **The 5000 random genes**. Additional file 5 is a text file and describes the 5000 randomly selected background genes that were used to calculate the coexpression specificity score. These additional files are also available on our supplementary website [19]. Click here for file

## References

[B1] Davidson E (2001). Genomic Regulatory Systems: Development and Evolution.

[B2] Zhou Q, Wong WH (2004). CisModule: de novo discovery of cis-regulatory modules by hierarchical mixture modeling. Proc Natl Acad Sci U S A.

[B3] Aerts S, Van Loo P, Moreau Y, De Moor B (2004). A genetic algorithm for the detection of new cis-regulatory modules in sets of coregulated genes. Bioinformatics.

[B4] Sharan R, Ovcharenko I, Ben Hur A, Karp RM (2003). CREME: a framework for identifying cis-regulatory modules in human-mouse conserved segments. Bioinformatics.

[B5] Gupta M, Liu JS (2005). De novo cis-regulatory module elicitation for eukaryotic genomes. Proc Natl Acad Sci U S A.

[B6] Frith MC, Fu Y, Yu L, Chen JF, Hansen U, Weng Z (2004). Detection of functional DNA motifs via statistical over-representation. Nucleic Acids Res.

[B7] Van Loo P, Aerts S, Thienpont B, De Moor B, Moreau Y, Marynen P (2008). ModuleMiner - improved computational detection of cis-regulatory modules: are there different modes of gene regulation in embryonic development and adult tissues?. Genome Biol.

[B8] Matys V, Kel-Margoulis OV, Fricke E, Liebich I, Land S, Barre-Dirrie A (2006). TRANSFAC and its module TRANSCompel: transcriptional gene regulation in eukaryotes. Nucleic Acids Res.

[B9] Hertzberg L, Zuk O, Getz G, Domany E (2005). Finding motifs in promoter regions. J Comput Biol.

[B10] Zaki MJ, Hsiao C, Grossman R, Han J, Kumar V, Mannila H, Motwani R (2002). CHARM: An efficient algorithm for Closed Itemset Mining. Proceedings of the Second SIAM International Conference on Data Mining (SDM '02).

[B11] Boyer LA, Lee TI, Cole MF, Johnstone SE, Levine SS, Zucker JP (2005). Core transcriptional regulatory circuitry in human embryonic stem cells. Cell.

[B12] Wasserman WW, Sandelin A (2004). Applied bioinformatics for the identification of regulatory elements. Nat Rev Genet.

[B13] Tusher VG, Tibshirani R, Chu G (2001). Significance analysis of microarrays applied to the ionizing radiation response. Proc Natl Acad Sci U S A.

[B14] Kent WJ, Sugnet CW, Furey TS, Roskin KM, Pringle TH, Zahler AM (2002). The human genome browser at UCSC. Genome Res.

[B15] Aerts S, Van Loo P, Thijs G, Mayer H, de Martin R, Moreau Y (2005). TOUCAN 2: the all-inclusive open source workbench for regulatory sequence analysis. Nucleic Acids Res.

[B16] Coessens B, Thijs G, Aerts S, Marchal K, De Smet F, Engelen K (2003). INCLUSive: A web portal and service registry for microarray and regulatory sequence analysis. Nucleic Acids Res.

[B17] Thijs G, Marchal K, Lescot M, Rombauts S, De Moor B, Rouze P (2002). A Gibbs sampling method to detect overrepresented motifs in the upstream regions of coexpressed genes. J Comput Biol.

[B18] Gallo A, De Bie T, Cristianini N (2007). MINI: Mining Informative Non-redundant Itemsets. Proceedings of the 11th conference on Principles and Practice of Knowledge Discovery in Databases (PKDD07), Warsaw, September.

[B19] (2008). ModuleDigger: an itemset mining framework for the detection of cis-regulatory modules. http://homes.esat.kuleuven.be/~hsun/ModuleDigger/Index.html.

